# Kernel Based Nonlinear Dimensionality Reduction and Classification for Genomic Microarray

**DOI:** 10.3390/s8074186

**Published:** 2008-07-15

**Authors:** Xuehua Li, Lan Shu

**Affiliations:** School of Applied Mathematics, University of Electronic Science and Technology of China, Chengdu, 610054, P.R. China.

**Keywords:** Manifold learning, Dimensionality reduction, Locally linear embedding, Kernel methods, Support vector machine

## Abstract

Genomic microarrays are powerful research tools in bioinformatics and modern medicinal research because they enable massively-parallel assays and simultaneous monitoring of thousands of gene expression of biological samples. However, a simple microarray experiment often leads to very high-dimensional data and a huge amount of information, the vast amount of data challenges researchers into extracting the important features and reducing the high dimensionality. In this paper, a nonlinear dimensionality reduction kernel method based locally linear embedding(LLE) is proposed, and fuzzy K-nearest neighbors algorithm which denoises datasets will be introduced as a replacement to the classical LLE's KNN algorithm. In addition, kernel method based support vector machine (SVM) will be used to classify genomic microarray data sets in this paper. We demonstrate the application of the techniques to two published DNA microarray data sets. The experimental results confirm the superiority and high success rates of the presented method.

## 1 Introduction

The recent sequencing of the human genome has opened a new era in biomedical research; genomic microarray data have attracted a great deal of attention, as reflected by the ever increasing number of publications on this technology in the past decade. The application of microarrays technology encompasses many fields of study. From the search for differentially expressed genes, genomic microarrays data present enormous opportunities and challenges for machine learning, data mining, pattern recognition, and statistical analysis, among others. In particular, microarray technology is a rapidly maturing technology that provides the opportunity to assay the expression levels of thousands or tens of thousands of genes in a single experiment [1]. Nevertheless, microarrays experiments usually produce a huge amount of data and high dimensionality in relatively small sample sizes (commonly on the order of tens or hundreds). Hence, the biggest challenge of microarrays experiments is data mining and dimensionality reduction. Manifold learning is a perfect tool for data mining that discovers the structure of high dimensional data sets and provides better understanding of the data. Several different manifold learning algorithms have been developed to perform dimensionality reduction of low-dimensional nonlinear manifolds embedded in a high dimensional space. Isomap [2], LLE [3], Laplacian eigenmaps, and Stochastic neighbor embedding were originally proposed as a generalization of multidimensional scaling.

The LLE is considered as among one of the most effective dimensionality reduction algorithms for data preprocessing of high-dimensional data and streaming, and has been used to solve various problems in information processing, pattern recognition, and data mining [4–6]. LLE algorithm computes a different local quantity, and calculates the best coefficients to approximate each point by a weighted linear combination of its neighbors, and then tries to find a set of low-dimensional points, which can be linearly approximated by its neighbors with the same coefficients that have been determined from high-dimensional points. However, when LLE is applied to real world datasets and applications, it displays limitations, such as sensitivity to the noise, outliers, missing data, and poor linear correlation between variables due to poorly distributed variables. In LLE algorithms, the free parameter is the LLE's neighborhood size, which unfortunately, has no direct method of finding the optimal parameter. The optimal neighborhood size for each problem is determined by the experimenter's experience. On the other hand, if the density of training data is uneven, it will decrease the precision of classification if only the sequence of first *k* nearest neighbors is considered and not the differences of distances.

The purpose of this paper is to fill these gaps by presenting a kernel method based LLE algorithm(KLLE). The kernel method [7, 8] is demonstrated as having the ability to extract the complicated nonlinear information from application datasets. The kernel function of the kernel method is a nonlinear mapping from input space 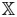 ⊆ ℜ*^n^* onto feature space ℍ ⊆ ℜ*^N^*, *φ*: 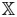 ⊆ ℜ*^n^* → ℍ ⊆ ℜ*^N^*. The kernel method provides a powerful and principled way of detecting nonlinear relations using well-understood linear algorithms in an appropriate feature space. This approach decouples the design of the algorithm from specification of the feature space. Most importantly, based on the kernel method, the kernel matrix is guaranteed to be positive semi-definite, convenient for the learning algorithm receiving information about the feature space and input data, and projects data onto an associated manifold, such as PCA. In addition, to solve KNN's parameter problems, fuzzy KNN adopts the theory of fuzzy sets to KNN, and fuzzy KNN assigns fuzzy membership as a function of the object's distance from its K-nearest neighbors and the memberships in the possible classes. This combination has two advantages. Firstly, fuzzy KNN can denoise training datasets. And secondly, the number of nearest neighbors selection, though not the most important, can consider the neighbor's fuzzy membership value.

Recently, support vector machine(SVM) has been extensively used by the machine learning community because it effectively deals with high dimensional data, provides good generalization properties, and defines the classifier architecture in terms of the so-called support vectors [8]. The theory of SVM is based on the idea of structural minimization, which shows that the generalization error is bounded by the sum of the training set and a term depending on the Vapnik-Chervonenkis dimension. By minimizing this bound, high generalization performance can be achieved. Moreover, unlike other machine learning methods, SVM generalization error is not related to the problem's input dimensionality.

This paper focused on genomic microarray analysis, which enables researchers to monitor the expression levels of thousands of genes simultaneously [9]. With the help of gene expressions, heterogeneous cancers can be classified into appropriate subtypes. To classify tissue samples or diagnose diseases based on gene expression profiles, both classic discriminant analysis and contemporary classification methods have been used and developed. Recently, different kinds of machine learning and statistical methods [10, 11] have been used to classify cancers using genomic microarrays expression data. To evaluate the effectiveness of the proposed KLLE dimensionality reduction method for classification, two published datasets are used. The experiment shows that dimensionality reduction of genes can significantly increase classification accuracy.

The remainder of this paper is organized as follows. In Section 2, we introduce the kernel method. The kernel method based LLE algorithm is constructed in Section 3. In Section 4, the kernel method based SVM is introduced. In section 5, we apply our proposed dimensionality reduction method to the Lymphoma and the SRBCT genomic microarray data sets, experiments and comparisons are conducted and presented. Conclusions are drawn in the final section.

## 2 Summary of Kernel Method

The kernel method [7] has become one of the most popular approaches to learning from examples with many potential applications in science and engineering [12]. The kernel method has been demonstrated to be able to extract the complicated nonlinear information embedded on a dataset. Many algorithms for data analysis are based on the assumption that the data can be represented as vectors in a finite dimensional vector space, such as linear discrimination, PCA, or least squares regression, making extensive use of the linear structure. Roughly speaking, the kernel method allows natural derivations of nonlinear versions. The general idea is described as follows. Given a linear algorithm (i.e., an algorithm which works in a vector space), one first maps the data living in a space 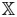 (the input space) to a vector space ℍ (the feature space) via a nonlinear mapping *ψ*: 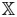 ⊆ ℜ*^n^* → ℍ ⊆ ℜ^N^, the kernel function is the form *K*(*x_i_*, *x_j_*) = 〈*ψ* (*x_i_*), *ψ* (*x_j_*)〉, and the kernel matrix is *K* = (*K_ij_*) = (*K*(*x_i_, x_j_*)), respectively. Then, linear algorithms may be applied to the vector representation *ψ*(*x*) of the data, which performs nonlinear analysis of data by linear method. In other words, the kernel method is an attractive computational shortcut, the purpose of the mapping *ψ*(·) is to translate nonlinear structures of data into new linear representation in ℍ.

The kernel methods solution comprises two parts: a module that performs the mapping into the embedding or feature space and a learn algorithm designed to discover linear patterns in that space. Firstly, we need to create a complicated linear feature space, and then work out what the inner product in that space would be, and finally find a direct method for computing that value in terms of the original inputs. In fact, the kernel function *K* is directly defined by the nonlinear mapping *ψ*(·), and the feature space ℍ is simply derived from its definition. The main property of kernel function is that the fundamental concept of the kernel method is the deformation of the vector (lower) space itself to a higher dimensional space. In general, a higher dimension linear space is clearer to classify than a low dimension one.

However, an explicit mapping *ψ* (·) does not always exist, and kernel method's conditions are not sufficient in guaranteeing the existence of a feature space. In practice, the mapping is performed implicitly by choosing a suitable kernel function *K*(*x_i_*, *x_j_*) = 〈*ψ*(*x_i_*), *ψ*(*x_j_*)〉 for the data points *x_i_* and *x_j_* . Moreover, there is a problem when choosing the function K(*x_i_*, *x_j_*), since not every function is guaranteed to give a valid feature space. One way of searching for a valid kernel function is to draw on Mercer's theorem [13] which states that any continuous symmetric function *K*(*x_i_*, *x_j_*) that satisfies the positive semi-definite condition

(1) $$\begin{array}{lll} {\int \;\int }_{𝕏\times 𝕏}\;K\;(x_{i},x_{j}\;)\psi \;(x_{i}\;)\psi \;(x_{j}\;)dx_{i}dx_{j}\geq 0 & \text{and} & \int_{𝕏}\psi {\;(x\;)}^{2}dx<\infty \end{array}$$

which is ensured to be a kernel for some valid feature space. This provides a flexible way of choosing the kernel mapping functions.

The kernel matrix is taken as an information bottleneck, which follows from the fact that the learning algorithm can glean from the training data and the chosen feature space is contained in the kernel matrix. The kernel matrix is not only the central concept in the design and analysis of the kernel method, but can also be regarded as the central data structure in their implementation. It is perhaps not surprising that some properties of the kernel matrix can be used to assess the performance of a learning algorithm.

## 3 Kernel Method based LLE Algorithm for Dimensionality Reduction

### 3.1 Locally Linear Embedding

LLE [3] is a manifold learning method that has aroused a great deal of interest in machine learning. It computes low-dimensional, neighborhood-preserving embeddings of high-dimensional inputs and recovers the global nonlinear structure from locally linear fits. Essentially, the algorithm attempts to compute a low dimensional embedding with the property that nearby points in the high dimensional space remain nearby and similarly co-located with respect to one another in the low dimensional space. Put another way, the embedding is optimized to preserve the local configurations of nearest neighbors. LLE computes dimensionality reduction that preserves the local neighborhood structure of the input data in the low-dimensional transformation. The transformation models the subspace manifold as a connected patchwork of locally linear surfaces. LLE is commonly justified using Taylor's theorem which states that any differentiable function is linear at the limit in a small area around a point. LLE works by identifying local neighborhood distance relationships, and by finding a mapping into a lower dimensionality that preserves them as much as possible. The selection of *k* value is the key to dimensionality reduction. There have been numerous papers [14, 15] suggesting that the selection of the neighborhood number *k* is important to the original LLE. If the number *k* is larger, the algorithm will ignore or even lose the local nonlinear features on the manifold, just as the traditional PCA performs. In contrast, if the number *k* is defined as smaller, LLE will split the continuous manifold into detached locality pieces, because the global characteristics are lost. On the other hand, it is well known that LLE is sensitive to noise, and LLE cannot preserve well the local geometry of the data manifolds in the embedding space when there are outliers in the data. In general, the practical input data sets are usually contaminated by noise which are caused by the disturbance and measurement, and the data sets are nonlinear correlation. Thus, the selection of the neighborhood number is difficult in real application to datasets.

### 3.2 Fuzzy K-Nearest Neighbor Algorithms

Conventional fuzzy KNN algorithm assigns an unlabeled pattern *x* to the class which appears the most among its *k* nearest labeled neighbors. The algorithm is described as follows. The problem of classifying *N* entities into *M* classes can be formulated as *C* = {*c*_1_, c_2_,…, *c_M_*}, where *c_i_* denotes the *i*th class. The available information is assumed to be in a training data set Ω = { (*x*_1_, *c_i_*), (*x*_1_, *c_k_*),…., (*x_n_*, *c_j_*)} of n patterns *x_i_* and their corresponding class labels *c_i_* taking values from *C*. The KNN [16] rule is well known in the pattern recognition literature. According to the rule, an unclassified pattern *x* is assigned to the class represented by a majority of its *k* nearest neighbors in *Ω*. [17] proposed a new approach by combining the fuzzy set theory with KNN algorithm, and named it as the fuzzy KNN classifier algorithm. According to his approach, rather than individual classes as in KNN, the fuzzy memberships of samples are assigned to different categories according to the following formulation

(2) $$\mu_{i}\;(x\;)=\frac{\sum_{i=1}^{k}\mu_{ij}{\left\|x-x_{j}\right\|}^{2/m-1}}{\sum_{i=1}^{k}{\left\|x-x_{j}\right\|}^{2/m-1}}$$

where *k* is the number of nearest neighbors, *m* determines how heavily the distance is weighted when calculating each neighbor's contribution to the membership value, *μ_i_*(*x*) denotes the membership of the test pattern *x* to class *i*, ‖*x* − *x_j_*‖ is the distance between the test pattern *x* and its nearest training samples *x_j_*. In this paper, the Euclidean metric is used, and *μ_ij_* is the fuzzy membership value of the *j*th neighbor to the *i*th class. After calculating all the memberships for a query sample, it is assigned to the class with which it has the highest membership value. Fuzzy KNN algorithms have two main advantages over the traditional KNN algorithms. Firstly, while determining the class of the current residue, the algorithm is capable of taking into consideration the ambiguous nature of the neighbors if any. The algorithm has been designed such that these ambiguous neighbors do not play a crucial role in the classification of the current residue. The second advantage is that residues are assigned a membership value in each class rather than binary decision of 'belongs to' or 'does not belong to'. The advantage of such assignment is that these membership values act as strength or confidence with which the current residue belongs to a particular class.

### 3.3 Kernel Method based LLE Algorithm

The KLLE extends LLE to work with the kernel method, which is used to map the nonlinear data into the linear feature space, which best reconstructs it as a linear combination of its neighbors. Moreover, the kernel matrix is positive semi-definite, and some properties and eigen-decomposition of kernel matrix are used to optimize the KLLE's objective function. In another way, the larger candidate neighborhood number *k* is selected over the original LLE, and fuzzy KNN is used to calculate all the fuzzy memberships for the candidate neighborhood. It is then assigned to the new neighborhood set when it has the higher membership value, and the new neighborhood number *k̄* is obtained. The major modifications of KLLE to the original LLE algorithm are discussed below.

Step 1. Mapping. Let 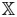 = {*x*_1_, *x*_2_, …, *x*_n_} be a set of *n* points in a high-dimensional data space ℜ*^D^*. Suppose that the space 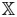 is mapped into a Hilbert space ℍ through a nonlinear mapping function *ψ:* 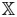
⊆ ℜ*^D^* → ℍ ⊆ ℜ*^N^*.
Step 2. The fuzzy neighborhood for each point. Assign neighbors to each data point *ψ*(*x_i_*) using the Fuzzy KNN algorithm. The *k̅* closest neighbors $\left\{\psi \;(x_{i}^{j}\;);j=1,\ldots ,\bar{k}\right\}$ are selected using the new define fuzzy Euclidean distance measure ${\left\|\psi \;(x_{i}^{j}\;)-\psi \;(x_{i}\;)\right\|}_{F}$, as follows:
  (3) $${\left\|\psi \;(x_{i}^{j}\;)-\psi \;(x_{i}\;)\right\|}_{F}^{2}={\left[\psi \;(x_{i}^{j}\;)-\psi \;(x_{i}\;)\right]}^{T}V_{i}^{-1}\left[\psi \;(x_{i}^{j}\;)-\psi \;(x_{i}\;)\right]$$
  where *V_i_* is a fuzzy covariance matrix of the point *x_i_*, and *V_i_* is a symmetric and positive definite matrix, which specifies the shape of the clusters. The matrix *V_i_* is commonly selected as the identity matrix, leading to Euclidean distance and, consequently, to spherical clusters, and and *V_i_* is defined as
  (4) $$V_{i}=\frac{{\sum_{j=1}^{k}\mu_{ij}^{2}[\psi \;(x_{i}^{j}\;)-\psi \;(x_{i}\;)]\;[\psi \;(x_{i}^{j}\;)-\psi \;(x_{i}\;)]}^{T}}{\sum_{t=1}^{n}\mu_{ij}^{2}}$$
Step 3. The kernel method based manifold reconstruction error. The KLLE's reconstruction error is similar to those of LLE, which is measured by cost function:
  (5) $$J\;(W\;)=\sum_{i=1}^{N}\|\psi \;(x_{i}\;)-\sum_{j=1}^{\overset{-}{k}}{W_{ij}\psi \;(x_{i}^{j}\;)\|}^{2}$$
  Considering reconstruction weights $\sum_{j=1}^{n}W_{ij}=1$, the reconstruction error can be rewritten by
  (6) $$J\;(W\;)=\sum_{i=1}^{N}{\left\|\sum_{j=1}^{\bar{k}}\left[\psi \;(x_{i}\;)-W_{ij}\psi (x_{i}^{j}\;)\right]\;\right\|}^{2}=\sum_{i=1}^{N}{\left\|\sum_{j=1}^{\bar{k}}W_{ij}\left[\psi \;(x_{i}\;)-\psi \;(x_{i}^{j}\;)\right]\;\right\|}^{2}=\sum_{i=1}^{N}J\;(W_{i}\;)\;$$
  (7) $$J\;(W_{i}\;)={\left\|\sum_{j=1}^{\bar{k}}W_{ij}\left[\psi \;(x_{i}\;)-\psi \;(x_{i}^{j}\;)\right]\;\right\|}^{2}={\left\|QW_{i}\right\|}^{2}=W_{i}^{T}Q^{T}QW_{i}=W_{i}^{T}KW_{i}$$
  where $Q_{i}=\left[\psi \;(x_{i}\;)-\psi \;(x_{i}^{1}\;),\psi \;(x_{i}\;)-\psi \;(x_{i}^{2}\;),\ldots ,\psi \;(x_{i}\;)-\psi \;(x_{i}^{\bar{k}}\;)\right]$; it is obvious that *Q^T^Q* is a positive semi-definite matrix. Then K = *Q^T^Q* is defined as a kernel matrix. Hence Eq.(7) which is subjected to $W_{i}^{T}1=1$ can be cast as the following Lagrange formulation
  (8) $$L\;(W_{i}\;)=W_{i}^{T}KW_{i}-\lambda \;(W_{i}^{T}I1-1\;)$$
  where the solution of Eq.(8) is $W_{i}=K^{-1}1/1^{T}K^{-1}1$, *K* is a positive definite matrix, the eigendecomposition of *K* is of the form *K* = *U^T^*Λ*U*, then *W_i_* = *U^T^*Λ^−1^*U***1**/**1***^T^U^T^*Λ^−1^*U***1.** Hence, the reconstruction weights *W* are computed by kernel matrix's eigenvalues and eigenvectors.
Step 4. The kernel method computes low-dimensional embedding *Y*. In this step, KLLE is used to compute the best low-dimensional embedding *Y* based on the weight matrix *W* obtained.
  (9) $$\Phi \;(Y\;)=\sum_{i=1}^{N}\|y_{i}-\sum_{j=1}^{\overset{-}{k}}{W_{ij}y_{i}^{j}\|}^{2}=tr\;(Y^{T}MY\;)$$
  subject to the constraints $\sum_{i=1}^{N}y_{i}=0$ and $\frac{1}{N}\sum_{i=1}^{N}y_{i}^{T}y_{i}=I$. Where *M* = (*I* − *W*)*^T^*(*I* − *W*), in LLE algorithm, the LLE embedding is given by the *d* eigenvectors correspond to the *d* smallest non-zero eigenvalues of matrix *M* [18].

In this step, we propose a method to yield KLLE embedding. *M* is a positive definite matrix, which has a maximum eigenvalue λ_1_, and the smallest eigenvalue is **0** and the corresponding eigenvector is the uninform vector **1** = (1,1,…, 1)*^T^*. Since the other eigenvectors are orthogonal to **1** and their coefficient sum to 0. Defining matrix *N: N* = (λ_1_*I* − *M*), it is obvious that *N* a positive definite matrix. If we compute the eigen-decomposition of *N*, the leading eigenvector is **1**, and the coordinates of the eigenvectors 2, 3, ……, *d*+1 provide the KLLE embedding. Equivalently, defining a new kernel matrix *K*

(10) $$K=\;(I-11^{T}\;)N\;(I-11^{T}\;)=\;(I-11^{T}\;)\left[\lambda_{1}I-{\;(I-W\;)}^{T}\;(I-W\;)\right]\;(I-11^{T}\;)$$

then, the eigenvectors 1,2,……, *d* of *K* provide the KLLE embedding in ℜ*^d^*.

The KLLE algorithm finds some global coordinates *x_i_* in the kernel space, over the lower-dimensional manifold, that conserve the local relations between neighboring points in the original embedding space. Each individual coordinate is obtained only from local information within its neighborhood. The overall KLLE algorithm only involves searching for closest points and basic matrix manipulations by kernel method and kernel matrix.

## 4 Kernel Method based SVM Classifier

The original support vector machine can be characterized as a powerful learning algorithm based on recent advances in statistical learning theory [19]. SVM is a learning system that uses a hypothesis space of linear functions in a high-dimensional space, trained with a learning algorithm from optimization theory that implements a learning bias derived from statistical learning theory. SVM uses a linear model to implement nonlinear class boundaries by mapping input vectors nonlinearly into a high-dimensional feature space using kernels. SVM has recently become one of the most popular tools for machine learning and data mining and can perform both classification and regression.

Let *S* = { (*x*_1_, *y*_1_), (*x*_2_, *y*_2_),……, (*x_n_*, *y_n_*)} be a training set with input data, where *x_i_* ∈ ℜ *^n^* is the training data and corresponding binary class labels *y_n_* ∈ {−1, 1}. Let the weight and the bias of the separating hyperplane be *w* and *b*, respectively, and the SVM classifier is

(11) $$f\;(x_{i}\;)=\text{sgn}\;(w\varphi \;(x_{i}\;)+b\;)$$

where *φ* is a nonlinear function, which maps *x* the input space into a feature space, To separate the data linearly in the feature space, the decision function satisfies the following conditions and the optimization problem is

(12) $$\begin{array}{lll} \text{Minimize} & {\left\|w\right\|}^{2}=<w,w> & \\ \text{subject to} & y_{i}\left[w\varphi \;(x_{i}\;)+b\right]\geq 1, & i=1,2,\ldots \ldots ,n \end{array}$$

The objective of SVM is to maximize the margin of separation and minimize the training errors. The problem can then be transformed into the following Lagrange formulation

(13) $$\begin{array}{l} \text{maximize}\;L\;(\alpha \;)=\sum_{i=1}^{n}\alpha_{i}-\frac{1}{2}\sum_{i,j=1}^{n}y_{i}y_{j}\alpha_{i}\alpha_{j}K\;(x_{i},x_{j}\;)\\ \begin{array}{ccc} \text{subject to}\sum_{i=1}^{n}y_{i}\alpha_{i}=0 & \alpha_{i}\geq 0, & i=1,2,\ldots ..n \end{array} \end{array}$$

where *K*(*x_i_*, *x_j_*) = 〈*φ*(*x_i_*), *φ*(*x_j_*)〉 is a kernel function and satisfies the Mercer theorem. The Karush-Kuhn-Tucker(KKT) complementarity conditions [19] provide useful information about the structure of the solution. The conditions state that the optimal solutions α*, w*, b* must satisfy

(14) $$\begin{array}{cc} \alpha_{i}^{*}\left[y\;(w^{*}\varphi \;(x_{i}\;)+b^{*}\;)-1\right]=0, & i=1,2,\ldots .n. \end{array}$$

where the $\alpha_{i}^{*}$ are the solutions of the dual problem and are non-zero only for a subset of vectors $x_{i}^{*}$ called support vectors. Then the resulting SVM for function estimation becomes

(15) $$f\;(x\;)=\text{sgn}\;(\sum_{i=1}^{m}\alpha_{i}^{*}y_{i}K\;(x,x_{i}^{*}\;)+b\;)$$

where *m* is the number of support vectors. SVM technique, developed by Vapnik [20], is a powerful widely used technique for solving supervised biological classification problems due to its generalization ability [21]. In essence, SVM classifiers maximize the margin between training data and the decision boundary, which can be formulated as a quadratic optimization problem in a feature space. The subset of patterns that are closest to the decision boundary are called support vectors. More details about SVM could be found in Vapnik's [19, 21, 22] and other publications [23, 24].

## 5 Performance Evaluation

The wide use of microarrays is in classification-for example, the prediction of the phenotype of a biological sample based on its patterns of gene expression. The analysis of gene expression profiles, which serve as molecular signatures for cancer classification and identification of differentially expressed groups of genes, provides a high-level view of functional classes or pathways, and has become challenge and significantly affecting topics in bioinformatics research. In order to do this, one needs a 'training set' of samples that have well-defined phenotypic differences, and that can be used to generate reproducible profiles. There is a wide range of algorithms that have been used for classification, artificial neural networks [25], discriminant analysis [26], classification and regression trees, support vector machines, and a host of other applications. Essentially, each of these uses an original set of samples, or training set, to develop a rule that takes a new test sample from a test set and uses its expression vector sample, trimmed to a previously identified set of classification genes, to place this test sample into the context of the original sample set, thus identifying its class.

In this section, we evaluate the performance of our kernel based dimensionality reduction algorithms and classifier to two published DNA microarray data sets: one is small round blue cell tumors(SRBCTs) dataset, and the other is lymphoma dataset. The manipulation is described as follows, in two steps: The first step, applying KLLE to project the training sets from *D*-dimensional space to the embedded lower-dimensional *d*-dimensional space. In the next step, the new *d*-dimensional dataset will be classified by employing SVM and some comparisons as presented.

### 5.1 SRBCT Data

In the first series of computational experiments, we considered a data set on SRBCTs presented in the work of [27]. This study included data from a 6567 element array of the 88 samples, tested over a training set of 63 samples and 25 test samples. These data consisted of 63 samples categorized into four classes: 23 Ewing family of tumors(EWS), 20 rhabdomyosarcoma(RMS), 12 neuroblastoma(BL), and 8 non-Hodgkin lymphoma(NB) , which are represented by the expression values of 2308 genes with suspected roles in processes relevant to these tumors. The test set consists of 25 samples: 6 for EWS, 3 for BL, 6 for NB, 5 for RMS, and 5 non-SRBCTs: 2 normal muscle tissues and 3 cell lines including an undifferentiated sarcoma, an osteosarcoma, and a prostate carcinoma. The 96 genes were selected by [27] from total data set of 6567 genes by a method using artificial neural networks to best distinguish the four groups in question.

**Figure 1.**
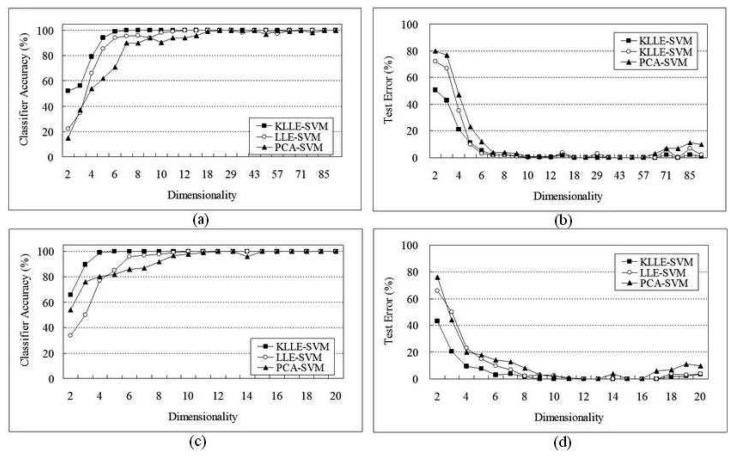
The experiments on the SRBCTs dataset: (a)The classifier accuracy of dimensionality reduction in 96 genes selected by Khan ;(b)The test error of dimensionality reduction in 96 genes; (c)The classifier accuracy of dimensionality reduction in 20 genes;(d)The test error of dimensionality reduction in 20 genes selected by Nikhil.

In this paper, considering some genes are irrelevant for diagnosis and would degrade the performance of the classifier, we followed Khan's and [28]'s methods for gene selection. We performed KLLE on the Khan's data set, consisting of expression levels of 96 genes, and the Nikhil's data set, only 20 genes based on FSMLP with online gene selection. In order to evaluate the dimensionality reduction, the comparisons between KLLE and principal component analysis(PCA) are done in the experiments. PCA is a standard dimensionality reduction tool, one rotates gene space, such that the variance is dominated by as few linear combinations as possible. KLLE, LLE and PCA were used to reduce the inputs to the dominant for classification. Furthermore, Gaussian kernel SVM is adopted as classifiers, and the kernel function in the analysis with parameters *σ* = 0.16 and *C* = 4. Using these 96 genes selected by [27], Fig.1 (a) shows the SVM classifier accuracy of the 63 training samples, and Fig.1 (b) shows the test errors of the 25 test samples. Using these 20 genes based on FSMLP with online gene selection by Nikhil, Fig.1 (c) and Fig.1 (d) show the SVM classifier accuracy and test errors of 88 samples. And all samples which are reduced to low-dimensional by KLLE, LLE and PCA, respectively.

By our purposed method, the classifier accuracy was 100% when the 20 genes were reduced to at least 5 dimensionality space, and 96 genes were reduced to at least 7 dimensionality space. However, by LLE method, the classifier accuracy was 100% when the 20 genes were reduced to at least 9 dimensionality space, and 96 genes were reduced to at least 14 dimensionality space. Finally, PCA method, the classifier accuracy was 100% when the 20 genes were reduced to at least 11 dimensionality space, and 96 genes were reduced to at least 29 dimensionality space. The implementation (classifier accuracy was 100%) returned a ranked list in about 1307 sec for the SRBCTs, 1743 sec by LLE and 1933 sec by PCA, much faster than 4127 sec by SVM without dimensionality reduction. Table 1 reports the comparison of the four methods on the SRBCTs dataset.

In fact, although the previous studies showed that linear classifiers are good enough to achieve almost perfect classification [29], there have some reports that the worst performances of the PCA based solution conform the need to take into account also nonlinear structures particularly for the SRBCTs dataset. We learned that the kernel method is more effective for classifier problems because of lying on non-linear separable feature space shown in most of cases. Using the 5 dimensionality vectors which were reduced from 20 genes, the KLLE-SVM classifier was able to correctly classify 25 test examples. Zero error occured and no misclassified example in the blind test was identified; therefore, the result is comparable with the works of [11] and [29].

## 5.2 Lymphoma Data

The second data set includes samples originating from the lymphoma dataset[Alizadeh et al], which can be obtained from http://llmpp.nih.gov/lymphoma. In this data set, there are 42 samples derived from diffuse large B-cell lymphoma (DLBCL), 9 samples from follicular lymphoma (FL), and 11 samples from chronic lymphocytic lymphoma (CLL). The entire data set includes the expression data of 4026 genes. In this data set, a small part of the data is missing. A k-nearest neighbor algorithm was applied to fill the missing values [30].

To find the genes that contribute most to the classification, the T-test, which has been used in gene selection [31] was used is to measure how large the difference is between the distributions of two groups of samples. To select important genes using the T-test involves two steps. In the first step, a score based on the T-test is calculated for each gene. In the second step, all the genes are rearranged according to their T-scores, and so on. The gene with the largest T-score is put in the first place of the ranking list, followed by the gene with the second greatest T-score. In this paper, in the lymphoma dataset, the top 165 genes selected from the lymphoma dataset by T-test. Another gene selection on lymphoma data is the nearest shrunken centroids [32] which used 48 genes to give a 100% accurate classification.

**Figure 2.**
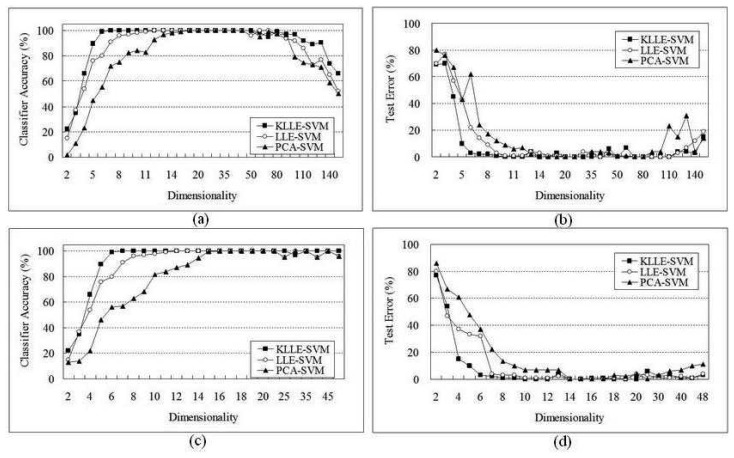
The experiments on the lymphoma dataset: (a)The classifier accuracy of dimensionality reduction in 165 genes selected by T-score;(b)The test error of dimensionality reduction in 165 genes; (c)The classifier accuracy of dimensionality reduction in 48 genes;(d)The test error of dimensionality reduction in 48 genes selected by nearest shrunken centroids

We followed the same procedure as we did in the SRBCT dataset. We performed KLLE, LLE, and PCA on the dataset which selected by T-score, consisting of expression levels of the top 165 genes, and the Tibshirani's data set, of which only 48 genes were selected based on the nearest shrunken centroids for gene selection. Fig.2 shows the classifier accuracy and the testing errors happened during classification. Using these 165 genes selected by T-test, Fig.2 (a) shows the SVM classifier accuracy of the training samples, and Fig.2 (b) shows the test errors of the test samples. Using these 48 genes selected by nearest shrunken centroids, Fig.2 (c) and Fig.2 (d) show the SVM classifier accuracy and test errors of the 42 samples, and all samples which are reduced to low-dimensional by KLLE, LLE and PCA, respectively.

For high-dimensionality reduced by the KLLE method, the KLLE-SVM classifier accuracy was 100% when the 48 genes were reduced to at least 7 dimensionality space, and 165 genes were reduced to at least 10 dimensionality space. However, by LLE method, the classifier accuracy was 100% when the 48 genes were reduced to at least 11 dimensionality space, and 165 genes were reduced to at least 15 dimensionality space. Finally, PCA method, the classifier accuracy was 100% when the 48 genes were reduced to at least 18 dimensionality space, and 165 genes were reduced to at least 22 dimensionality space. The implementation (classifier accuracy was 100%) returned a ranked list in about 1766 sec for the lymphoma dataset, 2247 sec by LLE, and 3105 sec by PCA, much faster than 5343 sec by SVM without dimensionality reduction. Table 2 reports the comparison of the four methods on the lymphoma dataset.

From the results it is obviously seen that KLLE performs excellently on datasets dimensionality reduction. Two facts demonstrate this capability. One the one hand, in nonlinear structures dataset, the kernel based nonlinear dimensionality reduction KLLE preserves intrinsic properties more than the linear LLE and PCA. On the other hand, we also found that the time consumption of KLLE-SVM is smaller than those of the other methods, than the time consumption of SVM in computing lower dimensionality. The results also show that our proposed KLLE enhances SVM competence of classification in high-dimensionality.

## 6 Conclusion

The application of machine learning to data mining and analysis in the area of microarray analysis is rapidly gaining interest in the community. The large number of gene expressions coupled with analysis over a time course, provides an immense space of genomic dimensionality reduction and selection. In this paper, we presented an effective approach to reduce high-dimensionality and genes classifier in genomic microarray experiments. In our approach, kernel method is demonstrated to be able to extract the complicated nonlinear information embedded on the data sets by a nonlinear mapping. This paper proposed an improved kernel locally linear embedding algorithm for dimensionality reduction, based on the traditional LLE, kernel method and fuzzy KNN. The proposed algorithm compresses and denoises the redundant information in manifolds and preserves most intrinsic properties at the same time. It is conformed that our proposed KLLE has overcome the some primary shortcomings of the original algorithm, stimulating the applications of LLE.

The experimental results indicate that the proposed method performs well in dimensionality reduction and achieve high classification accuracies in SRBCT and lymphoma dataset. And the results also showed that this approach preserved the dataset's intrinsic nonlinear relationship and performed better than the current popular LLE and PCA approach. We conclude that the KLLE not only helps biological researchers classify differentiate cancers that are difficult to be classified for high-dimensionality, but also helps researchers focus on a small number of important genes to find the nonlinear relationship between those important genes.

## Figures and Tables

**Table 1.** The comparison of three methods on SRBCT dataset

Algorithms	96 genes			20 genes		

	Dimensional	Support vectors	Time(sec)	Dimensional	Support vectors	Time(sec)
SVM	96	-	-	20	106	4127
PCA-SVM	29	87	2672	11	64	1933
LLE-SVM	14	63	2102	9	42	1743
KLLE-SVM	7	42	1934	5	31	1307

**Table 2.** The comparison of three methods on lymphoma dataset

Algorithms	165 genes			48 genes		

	Dimensional	Support vectors	Time(sec)	Dimensional	Support vectors	Time(sec)
SVM	165	-	-	48	124	5343
PCA-SVM	18	104	2672	22	83	3105
LLE-SVM	15	74	2133	9	56	2247
KLLE-SVM	7	56	1934	5	41	1766

## References

[1] Shalon D., Smith S.J., Brown P.O. (1996). A DNA microarray system for analyzing complex DNA samples using two-color fluorescent probe hybridization. Genome Research..

[2] Tenenbaum J.B., Silva V.de, Langford J.C. (2000). A global geometric framework for. nonlinear dimensionality reduction. Science..

[3] Roweis S.T., Saul L.K. (2000). Nonlinear Dimensionality Reduction by Locally Linear Embedding. Science..

[4] Zhang C., Wang J., Zhao N., Zhang D. (2004). Reconstruction and analysis of multi-pose face images based on nonlinear dimensionality reduction. Pattern Recognition..

[5] Elgammal A.M., Lee C.S. (2004). Separating style and content on a nonlinear manifold. IEEE Computer Society Conference on Computer Vision and Pattern Recognition..

[6] Mekuz N., Bauckhage C., Tsotsos J.K. (2005). Face recognition with weighted locally linear embedding. The Second Canadian Conference on Computer and Robot Vision..

[7] Schölkopf B., Smola A., Müller K.R. (1998). Nonlinear component analysis as a kernel eigenvalue problem. Neural Computation..

[8] Shawe-Talyor J., Cristianini N. (2004). Kernel Methods for Pattern Analysis..

[9] Young R.A. (2000). Biomedical discovery with DNA arrays. Cell..

[10] Brown M.P., Grundy W.N., Lin D., Cristianini N., Sugnet C.W., Furey T.S., Ares M.J., Haussler D. (2000). Knowledge-based analysis of microarray gene expression data by using support vector machines. Proc. Natl Acad. Sci..

[11] Lee Y., Lee C.K. (2003). Classification of multiple cancer types by multicategory support vector machines using gene expression data. Bioinformatics..

[12] Wang X.C., Paliwal K.K. (2003). Feature extraction and dimensionality reduction algorithms and their applications in vowel recognition, Pergamon. The Journal of the Pattern Recognition Society..

[13] Haykin S. (1999). Neural networks: A Comprehensive Foundation.

[14] Marina M., Shi J.b. (2001). Learning segmentation by random walks..

[15] Kouropteva O., Okun O., Pietikainen M. (2002). Selection of the optimal parameter value for the locally linear embedding algorithm. Proc of the 1st International Conference on Fuzzy Systems and Knowledge Discovery, Singapore.

[16] Cover T.M., Hart P.E. (1967). Nearest neighbour pattern classification. IEEE Trans. Inform. Theory..

[17] Keller J.M., Gray M.R., Givens J.A. (1985). A fuzzy k-nearest neighbours algorithm. IEEE Transactions on Systems, Man, and Cybernetics..

[18] Saul L., Roweis S. (2002). Think globally, fit locally: unsupervised learning of nonlinear manifolds. Technical Report MS CIS-02-18.

[19] Vapnik V.N. (1998). Statistical Learning Theory.

[20] Vapnik V.N. (1995). The nature of statistical learning theory..

[21] Qian Z., Cai Y.D., Li Y. (2006). A novel computational method to predict transcription factor DNA binding preference. Biochem. Biophys. Res. Commun..

[22] Vapnik V.N. (1999). An Overview of Statistical Learning Theory. IEEE Transactions on Neural Networks..

[23] Cristianini N., Shawe-Talyor J. (2000). An introduction to support vector Machines.

[24] Du P.F., He T., Li Y.D. (2007). Prediction of C-to-U RNA editing sites in higher plant mitochondria using only nucleotide sequence features. Biochemical and Biophysical Research Communications..

[25] Ellis M., Davis N., Coop A., Liu M., Schumaker L., Lee R.Y. (2002). Development and validation of a method for using breast core needle biopsies for gene expression microarray analyses. Clin. Cancer Res..

[26] Orr M.S., Scherf U. (2002). Large-scale gene expression analysis in molecular target discovery. Leukemia..

[27] Khan J., Wei J.S., Ringner M., Saal L.H., Ladanyi M., Westermann F. (2001). Classification and diagnostic prediction of cancers using gene expression profiling and artificial neural networks. Nat Med..

[28] Nikhil R.P., Kripamoy A., Animesh S., Amari S.I. Discovering biomarkers from gene expression data for predicting cancer subgroups using neural networks and relational fuzzy clustering. BMC Bioinformatics..

[29] Yeo G., Poggio T. (2001). Multiclass classification of SRBCTs. Technical Report AI Memo 2001-018 CBCL Memo 206, MIT..

[30] Troyanskaya O., Cantor M. (2001). Missing value estimation methods for DNA microarrays. Bioin-formatics..

[31] Tusher V.G., Tibshirani R., Chu G. (2001). Significance analysis of microarrays applied to the ionizing radiation response. Proc. Natl. Acad. Sci. USA..

[32] Tibshirani R., Hastie T., Narasimhan B., Chu G. (2003). Class predicition by nearest shrunken centroids with applications to DNA microarrays. Statistical Science..

